# Inactivation of the PD-1-Dependent Immunoregulation in Mice Exacerbates Contact Hypersensitivity Resembling Immune-Related Adverse Events

**DOI:** 10.3389/fimmu.2020.618711

**Published:** 2021-01-27

**Authors:** Matin Dokht Ashoori, Kensuke Suzuki, Yosuke Tokumaru, Naoko Ikuta, Masaki Tajima, Tasuku Honjo, Akio Ohta

**Affiliations:** ^1^Department of Immunology, Foundation for Biomedical Research and Innovation at Kobe, Kobe, Japan; ^2^Department of Immunology and Genomic Medicine, Graduate School of Medicine, Kyoto University, Kyoto, Japan; ^3^Pharmaceutical Research Labs, Meiji Seika Pharma Co., Ltd., Yokohama, Japan

**Keywords:** PD-1, cancer immunotherapy, immune-related adverse events, contact hypersensitivity, skin, T cells, CXCR3

## Abstract

Blockade of PD-1, an indispensable physiological immunoregulatory mechanism, enhances immune activities and is widely used in the immunotherapy of cancer. This treatment often accompanies inflammatory complication called immune-related adverse events (irAE), most frequently in the skin. To analyze how skin inflammation develops by the blockade of PD-1-dependent immunoregulation, we studied the exacerbation of oxazolone-induced contact hypersensitivity by PD-L1 blockade. The inactivation of PD-1 signaling enhanced swelling of the skin with massive CD8^+^ T cell infiltration. Among PD-1-expressing cells, T cells were the predominant targets of anti-PD-L1 mAb treatment since PD-L1 blockade did not affect skin inflammation in RAG2^-/-^ mice. PD-L1 blockade during immunization with oxazolone significantly promoted the development of hapten-reactive T cells in the draining lymph nodes. The enhancement of local CD8^+^ T cell-dominant immune responses by PD-L1 blockade was correlated with the upregulation of CXCL9 and CXCL10. Challenges with a low dose of oxazolone did not demonstrate any significant dermatitis; however, the influence of PD-L1 blockade on T cell immunity was strong enough to cause the emergence of notable dermatitis in this suboptimal dosing, suggesting its relevance to dermal irAE development. In the low-dose setting, the blockade of CXCR3, receptor of CXCL9/10, prevented the induction of T cell-dominant inflammation by anti-PD-L1 mAb. This experimental approach reproduced CD8^+^ T cell-dominant form of cutaneous inflammation by the blockade of PD-L1 that has been observed in dermal irAE in human patients.

## Introduction

The immune system has its own regulatory mechanisms with which the intensity of immune responses is modulated at proper levels. Since some of these mechanisms are exceptionally important, the deficiency of one of such immunoregulatory mechanisms may not be compensated by all of the rest. PD-1 represents the indispensable immunoregulatory mechanisms that spontaneously downregulate immune responses. When PD-1-expressing T cells recognize antigen presented on MHC, PD-1 interacts with PD-L1 or PD-L2 on their counterpart and triggers an inhibitory signal that interrupts T cell receptor signaling (1). PD-1 expression is not detectable in most resting T cells, but it is notably induced upon activation. This expression pattern indicates PD-1’s function as a negative feedback mechanism in activated immune cells. The pathophysiological importance of PD-1 has been demonstrated in spontaneous pathogenesis of inflammatory diseases in PD-1^-/-^ mice. PD-1-deficiency causes various autoimmune disease-like symptoms in different organs (1–3).

The major role of the PD-1-mediated regulation in the immune system led to the successful development of cancer immunotherapy. Blockade of the immunosuppressive signaling using anti-PD-1 mAb promoted anti-tumor immune responses and dramatically improved treatment of cancer patients including those who are not responding well to conventional treatments (4–7). The immunopotentiation by PD-1 blockade is not tumor-specific since the treatment causes inflammatory complications outside tumors in the significant proportion of patients. Such immune-related adverse events (irAE) in cancer patients can happen in various organs and in different forms of inflammation, e.g., self-reactive T cells and autoantibodies. Severe forms of irAE, higher than or equal to grade 3, have been observed in 7% of patients who received PD-1 blockade treatment. The induction of severe irAE is more frequent in CTLA-4 blockade reaching approximately 20% of patients (8). Although it is not very frequent, some irAE is critically dangerous such as severe colitis, myocarditis, pneumonitis and pancreatitis (9, 10). Therefore, the management of irAE is of importance in cancer immunotherapy. Anti-inflammatory treatment is effective, but it should be used with care not to compromise anti-tumor immunity. Experimental models of irAE will offer a springboard for the development of treatments.

Cutaneous inflammation is the most common form of irAE (10, 11). Most cases of this form are not life-threatening, e.g., rash, alopecia, and vitiligo. However, severe forms of cutaneous irAE are also reported in Stevens-Johnson syndrome and toxic epidermal necrolysis after the treatment with anti-PD-1 or anti-PD-L1 antibodies (12–16). Histochemical examination of affected skins from cutaneous irAE patients demonstrated CD8^+^ T cells accumulation and apoptotic keratinocytes, suggesting T cell-mediated pathophysiology (17). Levels of perforin and granzyme B were also found to increase in cutaneous irAE. This clinical observation indicates that PD-1-dependent immunoregulation is crucial to cutaneous inflammation. Consistent with this idea, PD-1^-/-^ T cells intensified allogenic reaction in mice and developed severe dermatitis accompanying extensive lymphocytes infiltration in the dermis (18). PD-L1 expression in keratinocytes can prevent T cell-dependent pathogenesis of dermatitis (19, 20).

To study the exaggeration of cutaneous inflammation by the blockade of PD-1 pathway, we used a contact hypersensitivity (CHS) model in mice. CHS is inducible by repeated exposure to a hapten, which induces proinflammatory activities of antigen-specific T cells (21). Depletion of CD4^+^ and CD8^+^ T cells is known to strongly impair CHS response. Prior to massive T cell accumulation, capillary vasodilation and neutrophils infiltration take place in the early phase of CHS. Keratinocytes recruit dendritic cells and neutrophils through the action of cytokines, chemokines, and chemical mediators. These inflammatory responses by non-T cells cooperate with subsequent antigen-specific T cell responses in optimal CHS induction.

In this study, blockade of the PD-1 pathway using anti-PD-L1 mAb exaggerated CHS along with local CD8^+^ T cell accumulation. The current study shows that T cells are the primary target of PD-1 blockade treatment in the enhancement of cutaneous inflammation. Upregulation of chemokines such as CXCL9 and CXCL10 was involved in the exacerbation of inflammation. The effect of PD-L1 blockade on T cell immunity was strong enough to cause the emergence of T cell-dominant dermatitis in the skin exposed to a suboptimal dose of hapten.

## Materials and Methods

### Mice

Female C57BL/6JJmsSlc mice were purchased from Japan SLC Co. (Shizuoka, Japan). PD-1^-/-^ mice and RAG2^-/-^ mice with C57BL/6-background were bred in our animal facility. The animals were housed under specific pathogen-free conditions and used between 8 and 12 weeks of age. All experiments were conducted in accordance with the institutional animal care guidelines.

### Induction of CHS

Mice were sensitized by topical application of 1 mg oxazolone (4-ethoxymethylene-2-phenyl-2-oxazolin-5-one; Sigma, St. Louis, MO) in 20 µl acetone/olive oil (4:1 vol/vol) on the shaved abdominal skin. Seven days later, mice were anesthetized with isoflurane to allow topical application of 20–200 µg oxazolone (10 µl) on the back side of the ear. Challenges were repeated every other day on day 9 and 11. The ear thickness was evaluated 48 h after each challenge using a digital thickness gauge (#547-301; Mitutoyo Corp., Kawasaki, Japan). On day 13, the ears were harvested from euthanized mice for further analysis.

### Treatment With Antibodies

To block PD-L1, mice received i.p. injections of anti-mouse PD-L1 mAb (1-111A; 0.3 mg/mouse) immediately after the sensitization and on days 3, 7, 9, and 11. CD8^+^ and CD4^+^ T cells was depleted by injecting mice intraperitoneally with 0.2 mg of anti-CD8b (clone: 2.43; BioXCell, Lebanon, NH) and anti-CD4 (clone: GK1.5; BioXCell) mAbs starting one day before sensitization (day -1) and on day 3 and 6. This treatment routinely depleted >98% of target cells. To block CXCR3, mice were given i.p injections of 0.2 mg of anti-CXCR3 (clone: CXCR3-173; BioXCell) on day 6 and 9.

### Preparation of Ear Cells

The ears were minced into small pieces and were incubated for 2 h at 37°C in 10 ml digestion solution. The digestion solution is Iscove’s Modified Dulbecco’s Medium (Gibco, Grand Island, NY) containing collagenase D (1 mg/ml; Roche, Mannheim, Germany) and DNase I (0.1 mg/ml Roche, Mannheim, Germany). Digested ears were disrupted using Fisherbrand 150 handheld homogenizer, and cells were passed through a 70 μm cell strainer (Falcon, Durham, USA). After erythrocytes removal using ACK Lysis Buffer (Gibco), cells were resuspended in PBS. Total viable cell numbers were determined by means of trypan blue exclusion.

### Flow Cytometric Analysis

Ear cells were preincubated with truStain FcX anti-mouse CD16/32 mAb for 10 min at 4°C. The following antibodies were used in the analysis: APC-anti-mouse CD4 (clone: RM4-5), BV421-anti-mouse CD8 (clone: 53-6.7), BV421-anti-mouse PD-1 (clone: 29F.1A12), rat-anti-mouse CD11b-APC (clone: M1/70), FITC-anti-mouse CD45 (clone: 30-F11), BV711-anti-mouse CD11c (clone: N418), PE-anti-mouse F4/80 (clone: BM8), and APC-anti-mouse NK1.1 (clone:PK136). All the antibodies were from BioLegend (San Diego, CA). All FACS analyses were performed on LSRFortessa flow cytometer (BD Biosciences, San Jose, CA), and data were analyzed by using FlowJo software (Treestar, Ashland, TN).

### Tissue Histology

Ear samples were collected on day 13 and fixed in 10% formalin-PBS. Tissue samples were processed and proceeded for hematoxylin-eosin staining by Applied Medical Research Laboratory (Osaka, Japan).

### Adoptive T Cell Transfer

Wild-type and PD-1^-/-^ C57BL/6 mice were sensitized with oxazolone as described above, and the inguinal lymph node and spleen were isolated after 7 days. The mixture of lymph node and spleen cells were labeled with FITC-anti-CD4 or FITC-anti-CD8 mAbs (Biolegend) and subsequently with anti-FITC microbeads (Miltenyi Biotec, Auburn, CA). CD4^+^ and CD8^+^ T cells were purified using AutoMACS (Miltenyi Biotec). A mixture of 7x10^6^ CD4^+^ and 5x10^6^ CD8^+^ T cells, equivalent to the purified cell numbers from one donor mouse, were injected intravenously into RAG2^-/-^ mice. The recipient mice received a challenge with 0.2mg oxazolone immediately after T cell transfer.

### Oxazolone-Specific T-Cell Responses

Wild-type mice were sensitized with oxazolone, and the inguinal lymph nodes were obtained after 7 days. The isolated lymph node cells (6 x 10^5^ cells) were tested for their capacity to produce IFN-*γ* in response to 0.1 mg/ml oxazolone. After 5 days of culture in a 96-well flat-bottomed plate, IFN-*γ* levels in the supernatant were determined by ELISA (R&D Systems, Minneapolis, MN). Oxazolone-specific IFN-*γ* production was calculated by subtracting the spontaneous cytokine release.

### RNA Isolation and qPCR

Ear samples were stored in 0.6 ml RNAlater (Qiagen, Germantown, MD). Tissues were cut into small pieces, disrupted using a homogenizer, and were passed through a 70 μm cell strainer. After washing twice with PBS, RNA was extracted using RNeasy Mini Kit (Qiagen). The extracted RNA was reverse transcribed to cDNA using the PrimeScript II kit (Takara-bio, Kusatsu, Japan) according to the manufacturer’s instruction. Real-time PCR was performed with SSo Advanced Universal SYBR Green Supermix (Bio-Rad, Hercules, CA) using the CFX Connect Real-Time PCR Detection System (Bio-Rad). PCR was performed by an initial denaturation of 95°C for 3 min, followed by 40 cycles of 95°C for 15 s and 55°C for 30 s. SYBR green fluorescence was measured at the end of each extension step. Melting curve analysis was performed to check the specificity of the PCR products. All samples were run in duplicate and averaged after normalization using β-actin as a housekeeping gene. Relative expression was quantified using 2^-ΔΔCT^ calculation. Primer sequences were as follows: 5- TCTGCCATGAAGTCCGCTG -3 and 5- CAGGAGCATCGTGCATTCCT-3 for CXCL9; 5- GCCGTCATTTTCTGCCTCAT-3 and 5- GCTTCCCTATGGCCCTCATT-3 for CXCL10; 5- TTCACCACACTAAGGGGCTA -3 and 5- GCCACAGAGAGATGGTGTTC -3 for CCL19; and 5- ACTATTGGCAACGAGCGGTTC -3 and 5- GGATGCCACAGGATTCCATAC -3 for β-actin. To discriminate mRNA expression in CD45^+^ and CD45^-^ populations, ear cell suspension was labeled with FITC-anti-mouse CD45 mAb and anti-FITC microbeads as described above. CD45^+^ and CD45^-^ fractions were purified using AutoMACS by positive and negative selection, respectively.

### Statistical Analysis

Data represent mean ± SD. Statistical calculations were performed using Student’s t-test (unpaired two-tailed) for the comparison between two groups. For the multiple comparison of more than two groups, we preformed Tukey-Kramer test. Statistical significance was accepted for p values less than 0.05.

## Results

### T Cell-Dependent Exacerbation of CHS by Anti-PD-L1 mAb Treatment

**Figure 1:**
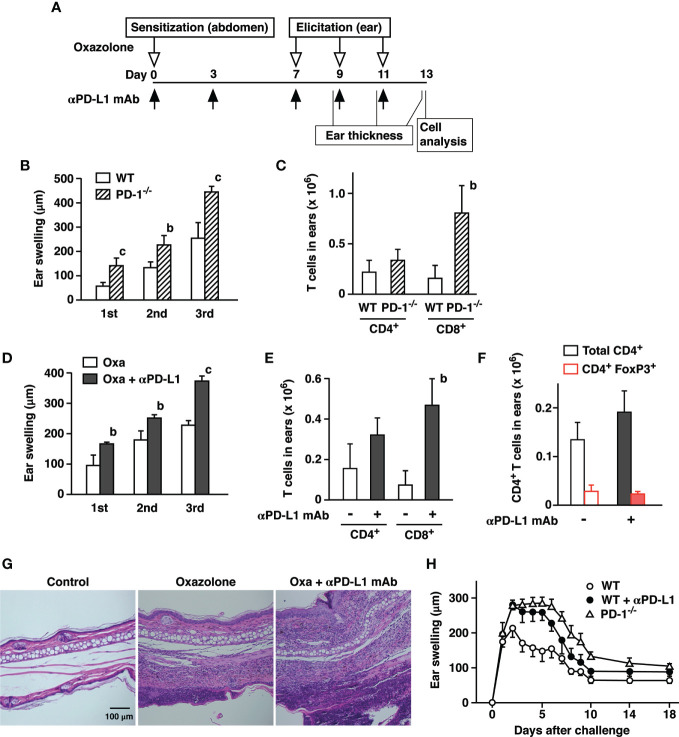
PD-1-dependent regulation of oxazolone-induced contact hypersensitivity. **(A)** Schedule of CHS induction. C57BL/6 mice were sensitized with oxazolone and received challenges at the ear on day 7, 9, and 11. Anti-PD-L1 mAb was given as indicated by black arrows. The extent of ear swelling is the ear thickness on day 9 (1st), 11 (2nd), and 13 (3rd) subtracted by the thickness before the challenge on day 7. **(B, C)** Challenges with oxazolone (200 µg) induced stronger skin inflammation in PD-1^-/-^ mice than in wild-type mice as indicated by the extent of ear swelling **(B)** and CD8^+^ T cell infiltration in the ears **(C)**. **(D–G)** Treatment of wild-type mice with anti-PD-L1 mAb enhanced ear swelling **(D)** and CD8^+^ T cell infiltration **(E)**, while the numbers of CD4^+^ T cells and CD4^+^ FoxP3^+^ cells remained at similar levels **(F)**. Tissue histochemistry **(G)** confirmed accumulation of inflammatory cells in the ear after 3-time challenge with oxazolone and further enhancement by anti-PD-L1 mAb treatment. **(H)** Time-dependent changes in ear swelling after one-time challenge with oxazolone. Data represent average ± SD of five mice. ^b^p < 0.01; ^c^p < 0.001 vs corresponding control groups (Student’s t-test).

Negative immunoregulation by PD-1 is so crucial to the immune system that its deficiency strongly augments inflammatory responses (1). We induced CHS in PD-1^-/-^ mice and compared the intensity with wild-type mice. To induce CHS, we first sensitized mice with oxazolone at the abdominal skin and challenged at the ear with the same hapten 7 days later (**Figure 1A**). The challenge with oxazolone was repeated every other day for 3 times. The extent of ear swelling was always greater in PD-1^-/-^ mice than in wild-type mice after each challenge (**Figure 1B**). We analyzed cell infiltrates in the ears two days after the third challenge and found an increase in CD8^+^ T cell accumulation in PD-1^-/-^ mice compared to wild-type mice (**Figure 1C**).

PD-L1 blockade in wild-type mice reproduced the same trend. Treatment with anti-PD-L1 mAb significantly enhanced ear swelling as well as the remarkable increase of CD8^+^ T cell accumulation in the ears (**Figures 1D, E**). The percentages of resident memory T cells defined as CD8^+^ CD103^+^ CD69^+^ were proportional in both groups and represented approximately 20–30% of CD8^+^ T cells. CD4^+^ T cells in the ear tissue showed an increasing trend, though the difference was not significant. FoxP3^+^ regulatory T cells accounted for 15–20% of CD4^+^ cells in both groups, and cell numbers remained at similar levels (**Figure 1F**). Histochemical examination of the affected ears confirmed massive infiltration of inflammatory cells in anti-PD-L1 mAb-treated mice (**Figure 1G**). Time-course of ear swelling after a single challenge showed that it is peaking after 2 days and slowly regressed thereafter before it reached a constant level on day 10 (**Figure 1H**). Inactivation of PD-1-dependent immunoregulation not only enhanced the intensity of swelling but also prolonged the peak levels for several more days.

**Figure 2:**
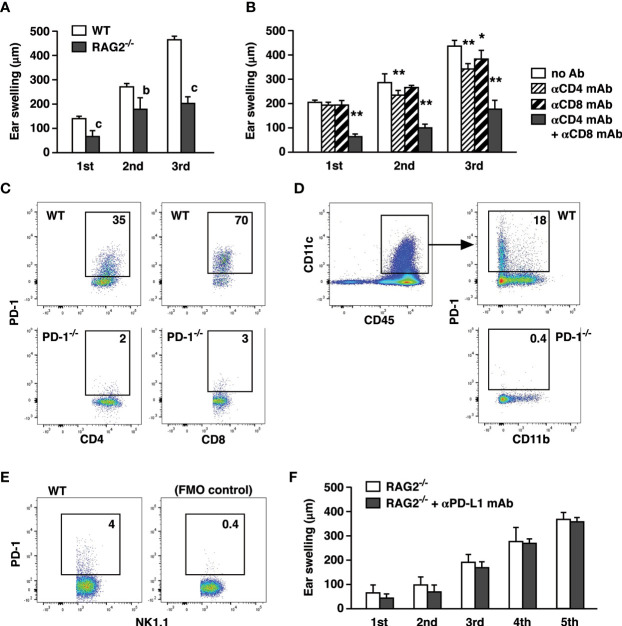
T cells are the principal targets of CHS enhancement by PD-L1 blockade. **(A)** CHS induction with 200 µg oxazolone in RAG2^-/-^ mice resulted in a significant but weaker ear swelling than wild-type mice. **(B)** T cell depletion in wild-type mice reduced the extent of CHS. Anti-CD4 and/or anti-CD8 mAbs were injected on day -1, 3, and 6. **(C–E)** PD-1 expression in the ear-infiltrated cells. Flow cytometric profiles indicate PD-1-positive percentage within CD4^+^, CD8^+^ **(C)**, CD11c^+^ **(D)**, and NK1.1^+^ **(E)** populations after gating for CD45^+^ events. CD4^+^, CD8^+^, and CD11c^+^ cells from PD-1^-/-^ mice were shown as negative controls. PD-1-positive NK1.1^+^ cells were indicated along with fluorescence-minus-one control. **(F)** Anti-PD-L1 mAb treatment did not exaggerate ear swelling in RAG2^-/-^ mice. Data represent average ± SD of five mice. ^b^p < 0.01; ^c^p < 0.001 vs corresponding control groups (Student’s t-test). *p < 0.05; **p < 0.01 vs no Ab group (Tukey-Kramer test).

CHS is generally recognized as a hapten-specific T cell-dependent inflammation, but T cell responses do not fully account for the inflammation in CHS. CHS in RAG2^-/-^ mice induced a significant but lesser degree of ear swelling than in wild-type mice (**Figure 2A**). Depletion of either CD4^+^ or CD8^+^ T cells had a moderate impact on CHS induction, but when both CD4^+^ and CD8^+^ T cells were depleted, ear swelling was greatly reduced to the levels observed in RAG2^-/-^ mice (**Figure 2B**). Although these results support T cell-dependence of CHS induction, ear swelling was still significant in the absence of T cells. It is consistent with previous papers regarding significant ear swelling by the CHS induction in RAG2^-/-^ mice. This CHS-like response in RAG2^-/-^ was mediated by NK cells in a hapten-specific manner (22, 23). Therefore, the blockade of PD-L1 might have exaggerated CHS by affecting T cell-dependent immune response and/or proinflammatory activities by innate immune cells.

PD-1 expression was originally discovered in activated T cells and B cells; however, more recently, NK cells and myeloid cells were also shown to express PD-1 (24–29). In our CHS induction, PD-1 expression could be found not only in T cells but also in NK cells and dendritic cells (**Figures 2C–E**). While PD-1 blockade is known to enhance T cell immunity, anti-PD-L1 mAb treatment might have found different targets other than T cells. To examine this possibility, we applied anti-PD-L1 mAb to CHS induction in RAG2^-/-^ mice. However, PD-L1 blockade did not enhance the ear swelling in RAG2^-/-^ mice at all even after the extended 5-time challenges with oxazolone (**Figure 2F**).

**Figure 3:**
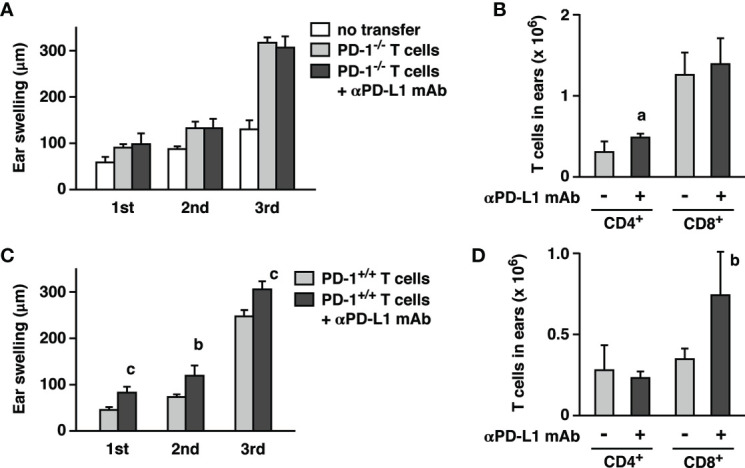
Adoptive transfer of CHS effector T cells into RAG2^-/-^ mice reconstituted immunopotentiation by anti-PD-L1 mAb. Effector T cells were obtained from oxazolone-sensitized wild-type **(C, D)** and PD-1^-/-^ mice **(A, B)**. Oxazolone dose for challenge was 200 µg. PD-L1 blockade enhanced ear swelling **(A, C)** and CD8^+^ T cell accumulation **(B, D)** when effector T cells were from wild-type mice, but not PD-1^-/-^ mice. Data represent average ± SD of five mice. ^a^p < 0.05; ^b^p < 0.01; ^c^p < 0.001 vs corresponding control groups (Student’s t-test).

Before ear-infiltrated T cells exert the proinflammatory activities, they need to interact with antigen-presenting cells for recognition of the hapten. It was still possible that PD-L1 blockade had somehow substantially changed T cell-stimulatory functions of PD-1-expressing antigen-presenting cells. Previous experiment with RAG2^-/-^ mice could not address such an indirect effect because they were lacking T cells. To test the significance of PD-1-expressing non-T cells in the presence of T cells, we injected PD-1^+/+^ or PD-1^-/-^ T cells to RAG2-KO mice and compared effects of anti-PD-L1 mAb on CHS. When PD-1^-/-^ T cells were used, PD-L1 blockade would not provide a direct benefit on T cells, but RAG2^-/-^ mice-derived non-T cells should be able to express PD-1. PD-1^-/-^ T cell transfer certainly promoted ear swelling on top of the innate response in RAG2^-/-^ mice, but anti-PD-L1 mAb failed to enhance CHS in this setting (**Figures 3A, B**). Transfer of PD-1^+/+^ T cells confirmed that anti-PD-L1 mAb is acting on T cells as observed in the further enhancement of ear swelling and CD8^+^ T cell number (**Figures 3C, D**). These results suggest that T cells are the primary target of CHS enhancement by anti-PD-L1 mAb. Although biological significance of PD-1 has been reported in NK cells and antigen-presenting cells (24–29), liberation of non-T cells from PD-1-dependent immunoregulation might have little contribution to the enhancement of CHS, if any.

### PD-L1 Blockade Promotes the Priming of Hapten-Reactive T Cells

**Figure 4:**
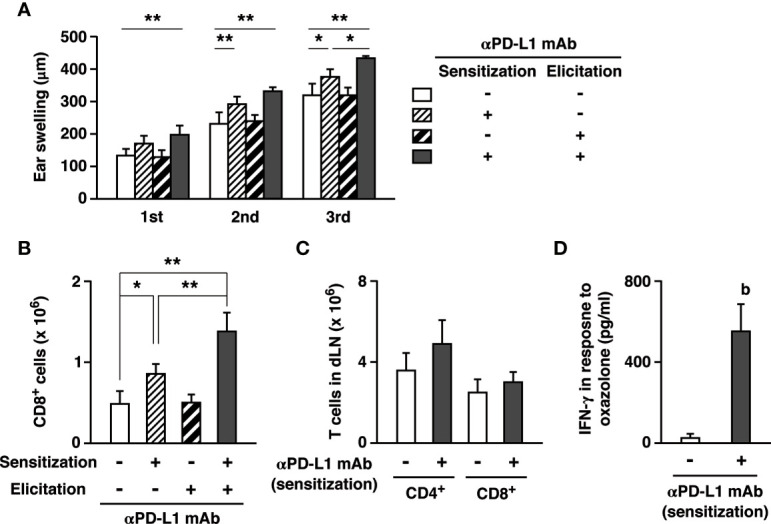
PD-L1 blockade could exaggerate CHS through the promotion of effector T cell development during sensitization. **(A, B)** Anti-PD-L1 mAb was given to the mice on either the sensitization phase (day 0 and 3) or the elicitation phase (day 7, 9, 11). Oxazolone dose for challenge was 200 µg. Ear swelling **(A)** and CD8^+^ T cell accumulation **(B)** by PD-L1 blockade in the sensitization phase were further enhanced by the extended anti-PD-L1 mAb treatment in the elicitation phase. **(C, D)** PD-L1 blockade during sensitization promoted the establishment of hapten-reactive T cells. The inguinal lymph nodes were analyzed 7 days after the sensitization on the abdomen. T cell numbers **(C)** did not change, but anti-PD-L1 mAb treatment during sensitization notably increased IFN-*γ* production in response to ex vivo restimulation with oxazolone (0.1 mg/ml) for 5 days **(D)**. Oxazolone-specific cytokine production was calculated as IFN-*γ* levels in the supernatant of oxazolone-stimulated lymph node cells subtracted by the levels in the unstimulated culture. Data represent average ± SD of five mice. Ex vivo restimulation of lymph mode cells were conducted in triplicate for each of three mice. ^b^p < 0.01 vs the control group (Student’s t-test). *p < 0.05; **p < 0.01 between indicated groups (Tukey-Kramer test).

CHS induction in this animal model consists of two different phases. The first is the sensitization phase where hapten application on dorsal or abdominal skin establishes hapten-reactive T cells. In the subsequent elicitation phase, a challenge with the same hapten at the ear recruits the hapten-reactive T cells and induce cutaneous inflammation. To examine which step is crucial to the immunopotentiation by PD-L1 blockade, we limited anti-PD-L1 mAb administration in either the sensitization phase or the elicitation phase. PD-L1 blockade only during the elicitation phase affected neither ear swelling nor T cell infiltration (**Figures 4A, B**). In contrast, when PD-L1 blockade was provided during the sensitization phase but withheld in the elicitation phase, the treatment sufficiently enhanced ear swelling and CD8^+^ T cell infiltration. Since PD-L1 blockade created a difference in the sensitization phase, we analyzed T cells in the inguinal lymph nodes after abdominal sensitization. T cell numbers in the lymph nodes did not significantly increase after the anti-PD-L1 mAb treatment (**Figure 4C**). To examine hapten-specific T cell response, we restimulated the lymph node cells with oxazolone and found a significant increase of IFN-*γ* production in anti-PD-L1 mAb-treated mice (**Figure 4D**). These results suggest that PD-L1 blockade could promote establishment of hapten-specific T cells in the sensitization, and the increase in hapten-specific T cells might be enough to enhance cutaneous inflammation.

Anti-PD-L1 mAb treatment in the sensitization phase alone promoted CHS, but interestingly, continuous PD-L1 blockade in both sensitization and elicitation phases further enhanced CHS (**Figures 4A, B**). This increase indicates that anti-PD-L1 mAb could also enhance inflammatory responses in the elicitation phase. In agreement with this view, PD-L1 blockade enhanced the elicitation phase of CHS in the transfer of effector T cells from oxazolone-sensitized mice to RAG2^-/-^ mice (**Figures 3C, D**).

### PD-L1 Blockade Enhances CXCR3-Dependent T Cell Accumulation

**Figure 5:**
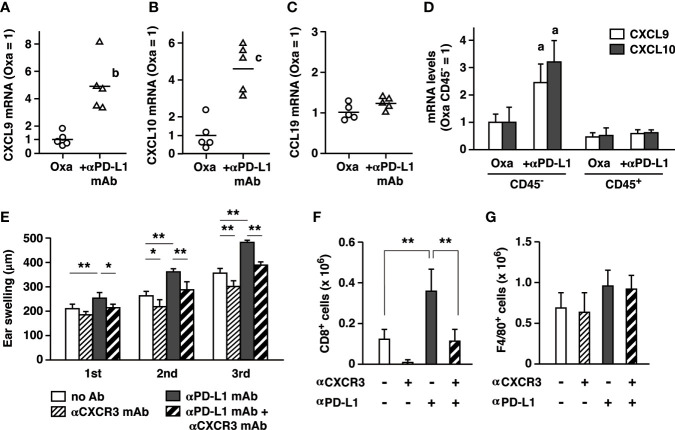
PD-L1 blockade enhanced CD8^+^ T cell accumulation by upregulating CXCL9 and CXCL10 in the inflamed ear. **(A–C)** mRNA levels of chemokines in the ear 24 h after the first challenge with oxazolone (200 µg). Anti-PD-L1 mAb treatment upregulated CXCL9 **(A)** and CXCL10 **(B)**, but not CCL19 **(C)**. **(D)** Upregulation of CXCL9 and CXCL10 was found in ear cells of non-hematopoietic origin (CD45^-^). mRNA levels were determined by qPCR using 5 **(A–C)** or 3 mice **(D)** per group. **(E–G)** Blockade of CXCR3 reduced the extent of swelling **(E)** and CD8^+^ T cell accumulation in the ear **(F)**. Anti-CXCR3 mAb (dose) was given on day 6 and 9. Blockade of PD-L1 or CXCR3 did not affect F4/80^+^ macrophages in the ear **(G)**. Data represent average ± SD of five mice. ^a^p < 0.05; ^b^p < 0.01; ^c^p < 0.001 vs corresponding control groups. *p < 0.05; **p < 0.01 between indicated groups (Tukey-Kramer test).

Treatment with anti-PD-L1 mAb increased numbers of inflammatory cells, especially CD8^+^ T cells, in the affected ears. Chemokines such as CXCL9 and CXCL10 have been shown to recruit CD8^+^ T cells to the inflamed tissues in disease models including allograft rejection and CHS (30–32). We examined whether these chemokines were responsible for the massive increase of CD8^+^ T cells by PD-L1 blockade in CHS. Anti-PD-L1 mAb treatment upregulated CXCL9 and CXCL10 mRNA in the inflamed ears (**Figures 5A, B**). mRNA levels of CCL19, which may attract dendritic cells and neutrophils *via* CCR7, did not increase by PD-L1 blockade (**Figure 5C**). In the ear, CD45^-^ cells of non-hematopoietic origin accounted for a large body of the chemokines induction by PD-L1 blockade (**Figure 5D**). Such PD-L1 blockade-dependent increases were not observed in the CD45^+^ fraction.

CXCR3 is a receptor for CXCL9 and CXCL10 and mediates immune cells recruitment to local inflamed sites. Blockade of CXCR3 significantly reduced ear swelling in the normal CHS induction and in the exaggerated CHS by anti-PD-L1 mAb (**Figure 5E**). Corresponding to this change, CD8^+^ T cell accumulation in the ear was largely decreased by anti-CXCR3 mAb treatment, confirming its central role in T cell recruitment to the local inflamed tissue (**Figure 5F**). In contrast, local macrophage counts did not change by anti-PD-L1 mAb or anti-CXCR3 mAb (**Figure 5G**). This experiment suggests that the pronounced CXCL9 and CXCL10 upregulation under PD-L1 blockade may be involved in the exaggerated cutaneous inflammation by promoting CD8^+^ T cell accumulation.

### PD-L1 Blockade Capitalizes on a Suboptimal Hapten Exposure to Induce Significant Dermatitis

**Figure 6:**
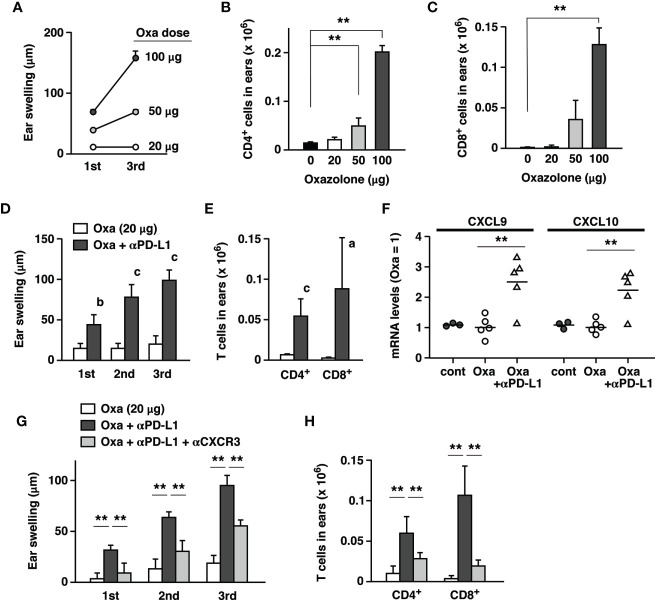
Exposure to a low dose of oxazolone did not demonstrate significant signs of dermatitis, but anti-PD-L1 mAb triggered remarkable tissue inflammation. **(A–C)** Dose-dependent CHS induction by oxazolone. A dose as low as 20 µg did not induce significant ear swelling **(A)** and T cell accumulation **(B, C)** by repeated challenges. **(D, E)** Although oxazolone (20 µg) alone did not induce significant inflammation, its combination with anti-PD-L1 mAb caused notable swelling **(D)** and T cell accumulation **(E)** in the ear. **(F)** Anti-PD-L1 mAb treatment upregulated CXCL9 and CXCL10 mRNA in the ears 24h after the challenge with 20 µg oxazolone. Oxazolone alone did not change the chemokine mRNA levels compared to untreated controls. **(G, H)** Anti-CXCR3 mAb suppressed the induction of era swelling **(G)** and T cell accumulation **(H)** by the suboptimal exposure to oxazolone (20 µg) plus PD-L1 blockade. Data represent average ± SD of 3 **(A–C)** or 5 **(D–H)** mice. ^a^p < 0.05; ^b^p < 0.01; ^c^p < 0.001 vs corresponding control groups (Student’s t-test). **p < 0.01 between indicated groups (Tukey-Kramer test).

The current study shows that distinct dermatitis in the hapten-induced CHS could be further exaggerated by the blockade of PD-1-dependent immunoregulation. However, irAE in PD-1 blockade therapy may be observed in tissues without previously recognizable inflammation. To reproduce this situation, we examined whether the immunopotentiation by anti-PD-L1 mAb was strong enough to escalate subtle inflammation to clearly visible dermatitis. We sought to determine a suboptimal dose of oxazolone and found that challenges with a dose as low as 20 µg did not demonstrate either significant tissue swelling or T cell accumulation in the ear (**Figures 6A–C**). There was no escalation of ear swelling even after the repeated challenges at this dose. However, when combined with PD-L1 blockade, exposure to this dose of oxazolone resulted in notable dermatitis, which was intensified by the repeated challenge (**Figure 6D**). Although a small number of T cells could be found in the ear with such a low dose of oxazolone, anti-PD-L1 mAb treatment increased CD4^+^ and CD8^+^ T cell numbers for 9- and 25-times (**Figure 6E**). This result suggests that the enhancement of T cell immunity by the blockade of PD-1 pathway can transform unnoticeable skin irritation to significant dermatitis.

The analysis of chemokines expression showed that anti-PD-L1 mAb treatment upregulated mRNA levels of CXCL9 and CXCL10 in the ears challenged with 20 µg oxazolone (**Figure 6F**). Anti-CXCR3 mAb downregulated ear swelling and T cell accumulation that were mostly caused by the anti-PD-L1 mAb treatment (**Figures 6G, H**). PD-L1 blockade promoted CD8^+^ T cell-dominant immune responses in the local skin and thereby facilitated the emergence of significant dermatitis.

## Discussion

PD-1-dependent immunoregulation plays a key role in cutaneous inflammation. In the current study, CHS induction in PD-1^-/-^ mice using oxazolone significantly enhanced tissue swelling as well as CD8^+^ T cell infiltration. Treatment of wild-type mice with anti-PD-L1 mAb also exaggerated dermatitis (**Figure 1**) as it has been shown in CHS experiments using dinitrophenylfluorobenzene as a hapten (33, 34).

Anti-PD-L1 mAb treatment during sensitization clearly increased oxazolone-reactive effector T cells in the draining lymph nodes (**Figure 4**). This result indicates that the PD-1 signaling critically controls the development of antigen-specific effector T cells in the lymph nodes. Promoted expansion of oxazolone-specific T cells could sufficiently enhance subsequent induction of CHS, even though anti-PD-L1 mAb treatment was withheld during the elicitation phase. PD-1-dependent immunoregulation was also crucial in the elicitation phase because extended treatment with anti-PD-L1 mAb into the elicitation phase further enhanced CHS (**Figure 4**). The enhancement of the elicitation phase by PD-L1 blockade was also evidenced from the T cell transfer experiment where the recipient mice received oxazolone challenge soon after the transfer of effector T cells from sensitized mice (**Figures 3C, D**). Consistent with these findings, PD-1-dependent immunoregulation has been shown to be vital in the elicitation phase of T cell-dependent cutaneous inflammation. PD-L1 expression in keratinocytes can reduce the intensity of skin inflammation (19, 20). Hapten-specific effector T cells are prone to PD-1-mediated inactivation since transfer of PD-L1-expressing dendritic cells to the sensitized mice downregulated CHS (35). CHS induction establishes tissue-resident CD8^+^ T cells in the skin, and PD-1 blockade in rechallenge augmented recall response of tissue-resident T cells in the enhanced dermatitis (34).

Interestingly, PD-1 blockade was previously reported to have no effect on CHS when applied only in the elicitation phase (33). Our data also showed no enhancement of inflammation when treatment with anti-PD-L1 mAb started 7 days after the sensitization (**Figure 4**). It is unclear why the treatment in the elicitation phase alone failed to enhance the inflammation, but it may be related to the numbers of hapten-reactive T cells. In the draining lymph nodes of sensitized mice without PD-L1 blockade, IFN-*γ* production in response to oxazolone was very little (**Figure 4**). Possibly, the number of oxazolone-reactive T cells after the conventional sensitization might be so small that immunopotentiation by PD-L1 blockade could not produce a significant impact in the subsequent elicitation phase. However, PD-1/PD-L1 blockade during the elicitation phase may significantly enhance the inflammation when a large number of hapten-specific effector T cells is available, e.g., vigorous induction with a help from PD-L1 blockade in the sensitization (Fig. 4) or effector T cell transfer into RAG2^-/-^ recipients (**Figure 3**). Multiple episodes of antigen exposure may enhance antigen-specific T cell immunity to the above threshold levels. Patients with such a history may be vulnerable to the emergence of irAE by PD-1/PD-L1 blockade.

The importance of T cells, especially CD8^+^ T cells, in CHS has been demonstrated in previous reports (36–39). In our experiment, depletion of CD4^+^ and CD8^+^ T cells from wild-type mice inhibited CHS induction and reduced ear swelling to the levels equivalent to those observed in RAG2^-/-^ mice (**Figure 2**). Although T cells are important players in CHS, the considerable ear swelling in the absence of T cells (**Figure 2**) indicates that non-T cells play a direct role in the cutaneous inflammation (21). In the original report on NK cell memory, CHS induction in RAG2^-/-^ mice demonstrated significant ear swelling in an antigen (hapten)-specific manner (22). Their study showed that NK cells were responsible for the cutaneous inflammation in RAG2^-/-^ mice because NK cell depletion abolished the ear swelling. This NK cell-dependent CHS involves unconventional ear inflammation with fewer cell infiltrates (23). Similar to the fellow lymphocytes, NK cells can express PD-1 after activation, and interaction of their PD-1 with PD-L1 downregulates NK activities (24–26). The significance of PD-1 in monocytes remains unclear, but it may be related to M2-type functions, and their anti-inflammatory nature may contribute to the immunosuppressive tumor microenvironment (29, 40). Along with T cells, these cell types were found to express PD-1 in the hapten-challenged ear (**Figure 2**). PD-L1 blockade on myeloid lineage or NK cells did not affect the intensity of dermatitis (**Figure 2**). The results from T cell transfer experiment excluded the possibility that PD-1-expressing non-T cells might indirectly exaggerate the inflammation by promoting T cell activity (**Figure 3**). These results strongly suggest that PD-1-expressing T cells, but not myeloid lineage or NK cells, are the predominant target of anti-PD-L1 mAb treatment in the enhancement of dermatitis.

Correlated with the remarkable increase of ear-infiltrated CD8^+^ T cells, PD-L1 blockade upregulated CXCL9 and CXCL10 (**Figure 4**). These chemokines are responsible for CD8^+^ T cell recruitment in various inflammation models (30–32) and in tumors (41, 42). CXCL9 and CXCL10 are induced in the normal course of CHS in mice (39, 43, 44) and humans (45), and their blockade can reduce CHS intensity (39, 46). CXCL9 and CXCL10 recruit T cells *via* CXCR3. Blockade of CXCR3 reduced T cell infiltration and attenuated ear swelling in anti-PD-L1 mAb-treated mice (**Figure 5**). Such a role of the CXCR3 chemokine system is consistent with tumor studies in which vigorous anti-tumor activities of CD8^+^ T cell response by the PD-1 blockade accompanies CXCL9/10 upregulation. CXCR3 blockade abolished intratumoral CD8^+^ T cell infiltration and anti-tumor efficacy of the PD-1 blockade therapy (41, 42). While anti-PD-L1 mAb targets T cells in the enhancement of CHS (**Figures 2**, **3**), significant upregulation of CXCL9 and CXCL10 was observed in CD45^-^ cells (**Figure 5**). The chemokine-producing non-hematopoietic cells might be keratinocytes, which has been shown to produce CXCL9 and CXCL10 in CHS (47–49). It is possible that PD-L1 blockade enhanced IFN-*γ* production from activated T cells, and thereby keratinocytes upregulated these IFN-*γ*-inducible chemokines (44, 50). Recently, PD-1-deficient CD8^+^ T cells were found to enhance CXCL9 expression in keratinocytes where the interaction with IFN-*γ*-producing T cells might have induced CXCL9 (51).

The exaggeration of CHS in this study and tumor regression in the immune checkpoint therapy are both initiated by the inactivation of PD-1 pathway; therefore, it is not surprising to find some similarities in between. As discussed above, the activation of CD8^+^ T cell-dominant immune response is important in both cases, and those T cells were recruited by CXCR3-chemokines. Another example is the promotion of antigen-specific T cell development as the mechanism of action. PD-L1 blockade during the sensitization with oxazolone promoted the induction of hapten-reactive T cells in the draining lymph nodes, and this increase was significant enough to enhance subsequent dermatitis induction (**Figure 4**). Correspondingly, surgical removal of tumor-draining lymph node abolished anti-tumor efficacy of anti-PD-L1 mAb (41). This finding indicates that the enhancement of effector functions of pre-existing tumor-infiltrated T cells might be insufficient but priming of anti-tumor effector T cells in the lymph nodes is crucial to achieve tumor regression by PD-1 blockade.

Experimental CHS induction in the presence of anti-PD-L1 mAb also shares an inflammatory profile with human irAE in the skin. Skin tissues of cancer patients who received PD-1 blockade therapy demonstrated notable CD8^+^ T cell infiltration and apoptotic keratinocytes (17). In accordance with the clinical observation, our experimental model showed characteristic CD8^+^ T cell-dominant infiltrates as the result of PD-L1 blockade. In these mice, PD-L1 blockade enhanced the upregulation of CXCL9/10 that can recruit CD8^+^ T cells. These chemokines have been also found to increase in patients’ skin. In addition to CXCL9/10, increases in perforin and granzyme B suggested proinflammatory activities of CD8^+^ T cells in patients’ skin. Such gene expression profiles in the skin from subjects of PD-1 blockade therapy resembled that of immune-related skin diseases such as Stevens-Johnson syndrome and toxic epidermal necrolysis (17). Indeed, although most skin problems related to PD-1/PD-L1-blocking agents are mild, those agents are reported to rarely induce severe irAE such as Stevens-Johnson syndrome and toxic epidermal necrolysis (12–16). Clinically, occurrence of irAE may be associated with anti-tumor efficacy of PD-1 blockade therapy. Improvement of progression-free survival has been reported in patients who experienced irAE including dermatological inflammation (52–54). This correlation seems to be reasonable because the same type of immune responses may be responsible for tumor regression and irAE induction as a result of PD-1/PD-L1 blockade.

Dermal irAE may arise in the previously asymptomatic skin as a result of immune checkpoint blockade therapy. Interestingly, minimum levels of skin irritation by a low dose of oxazolone were exaggerated to clearly visible dermatitis, suggesting that the proinflammatory effect of PD-L1 blockade had a great influence on dermatitis (**Figure 6**). Compared to the high dose setting, which represents pre-existing significant inflammatory conditions, this suboptimal setting may be closer to the clinical situation. Since a low dose of oxazolone alone hardly induced dermatitis (**Figure 6**), dermatitis in this setting was essentially caused by the proinflammatory effect of PD-L1 blockade. Although CHS is resulted from combined proinflammatory responses by T cells and innate immune cells, we have shown here that anti-PD-L1 mAb treatment predominantly promotes T cell-dependent immune response (**Figures 2**, **3**). Fold increase of accumulated T cell numbers were pronounced in the low-dose setting, suggesting that dermatitis caused by PD-1/PD-L1 blockade might involve more substantial contribution from T cell-dependent inflammation than the same levels of inflammation by conventional CHS induction. Therefore, dermatitis induction as the result of PD-1/PD-L1 blockade, i.e., dermal irAE, may be a relatively T cell-predominant form of inflammation. Antigens in clinical dermal irAE remain to be elucidated. Activation of immune response to antigens associated with tumor cells may trigger autoimmune T cell reaction to normal cells. Vitiligo has been linked to the activation of melanocyte antigen-specific T cells in the treatment of cancer including melanoma. Autoantibodies may be also implicated in the pathogenesis of irAE (10). In dermal irAE, autoantibodies to BP180/collagen XVII have been correlated with the development of irAE (55, 56). In our study, B cell infiltration in the inflamed ear remained unchanged after anti-PD-L1 mAb treatment and represented a minor population (3–4% of CD45^+^ cells); however, we cannot exclude a possibility that antibody production from B cells played a significant role in this inflammation.

In conclusion, the blockade of PD-1 signaling exaggerated CHS with massive CD8^+^ T cell infiltration. The mechanism involves the enhancement of antigen-specific T cell development and their accumulation into the local skin tissue through upregulation of CXCL9 and CXCL10. These changes in the CHS enhancement by PD-1 blockade shares features with clinical observations in dermal irAE, which shows CD8^+^ T cell-dominant form of cutaneous inflammation. Our experiment with a low dose of oxazolone indicates that the blockade of PD-1 pathway could substantiate a predisposing stealthy inflammation to prominent dermatitis. Inflammatory tissue damage in irAE is progressive in some patients and can persist even after the discontinuation of immunotherapy. Proper management of irAE is important to prevent its exacerbation; however, the use of anti-inflammatory treatment requires attention to its influence on tumor recurrence. This experimental approach may be useful in the analysis of dermal irAE and in developing treatment for this inflammatory complication.

## Data Availability Statement

The original contributions presented in the study are included in the article/supplementary materials. Further inquiries can be directed to the corresponding author.

## Ethics Statement

The animal study was reviewed and approved by Foundation for Biomedical Research and Innovation at Kobe.

## Author Contributions

TH and AO designed the study. MA, KS, YT, and MT performed the experiments and analyzed the data with AO. NI performed statistical analyses. AO wrote the first draft of the manuscript. MA contributed sections of the manuscript. All authors contributed to the article and approved the submitted version.

## Conflict of Interest

Authors KS and YT were employed by the company, Meiji Seika Pharma Co., Ltd.

The remaining authors declare that the research was conducted in the absence of any commercial or financial relationships that could be construed as a potential conflict of interest.
